# Bicycling for Women

**Published:** 1895-09

**Authors:** 


### *EDITORIAL.*

The office of THE JOURNAL is in room 330 Equitable Building.

The editors are not responsible for views expressed by contributors.

Articles for publication should reach this office not later than the 15th instant.

Address all communications, and make all remittances payable to THE ATLANTA MEDICAL AND SURGICAL JOURNAL, Atlanta, Ga.

Reprints of articles will be furnished, when desired, at a nominal cost. Requests for the same should always be made *on the manuscript*.

### BICYCLING FOR WOMEN.

The recent attack upon the female bicycle-rider by a well-known minister in this city in language more forcible than true or elegant, and the importance which the bicycle has assumed as a means of amusement and recreation to our girls and women justify some notice of the subject in this place.

The Rev. Dr. Hawthorne, who seems to be the self-appointed conservator and guardian of the morals and virtue of this community, has charged that the female bicyclist is immodest, indecent, unwomanly, mannish, etc.; and he has asserted with the positiveness of profound ignorance that the use of the wheel is highly injurious and productive of great damage to the health of the female rider. On this last point he claims to have the support of "many eminent specialists."

We believe that Dr. Hawthorne's knowledge of medicine is confined to the recommendation of his Royal Germetuer for every human ailment, from bee-sting to lockjaw; outside of that his opinion on matters medical is worth about as much as that of a sandfiddler on finance. Indeed, we doubt very much if the good doctor, in all his pompous wisdom, knows what an "eminent specialist" is. The one with his face in the paper, the self-styled "specialist" in "all diseases of men, women, and children," the medical blackleg, whose opinion on medical subjects is worth just a little more than Dr. Hawthorne's—he is probably the one the good doctor had in mind. But we let that pass, and return.

The statement as to the injuriousness of bicycle exercise for women is simply dogmatic and gratuitous assumption. There is no reason under the sun why the bicycle properly and intelligently used by women should not be healthful and invigorating, Dr. Hawthorne and his alleged specialists to the contrary notwithstanding. In the Boston *Medical and Surgical Journal* (June 13) Dr. Chas. W. Townsend discusses the subject from an enlightened standpoint. His conclusions are based upon the statements and experience of eighteen female physicians in and around Boston. These ladies and physicians, with one exception, stated decidedly that they considered bicycling of value to the average woman. One said: "My own personal experience leads me to consider bicycling a decidedly healthful, safe, agreeable, and cheap form of exercise." Most of them had observed no harm from the bicycle, and when this had occurred it was from excessive use of it. They had recommended the exercise in chronic uterine diseases where the circulation was sluggish and the muscles relaxed and weak. A number of cases were reported as benefited by the bicycle. Dr. Townsend concludes by saying that "bicycling is beneficial for women, not from any special effect on the pelvic organs, but because it is an agreeable, healthful form of exercise in the open air, a form which exercises the whole body, and indirectly benefits special conditions." In a late number of the New York *Journal of Obstetrics* Dr. Dickinson, of Brooklyn, says that "in biclycing there seems to have been found at last a form of outdoor muscular work which attracts women and entices them to many hours in the open air. It possesses all the advantages of walking or climbing, and exercises a large number of muscles. No single exercise will so develop muscular tone as this." And lastly we will add a clipping from the New York *Post-Graduate:* "The use of the bicycle has expanded and developed from a salutary athletic exercise into a great social obsession. It has seized upon every class of society, both sexes, all ages, and every condition of life. It is taken up by the well because it makes them feel better, by the invalid because it makes them feel well, by tired people because it rests them, and by the rested because it makes them feel tired. The fat ride to get thin and the thin to get fat. It has displaced the horse, and in women has, in a measure, replaced the uterus. It has made the simple and ancient custom of walking most unpopular, it has cut down the function of the steam-car, and competes successfully with the suburban trolley. The doctors have taken it up and expressed their approval of it, and we are far from saying a word in opposition. The bicycle has come to stay."

